# Temperature/pH Dual-Responsive Hydrogels: Research Progress in Preparation Methods, Structural Design Strategies and Biomedical Applications

**DOI:** 10.3390/gels12050433

**Published:** 2026-05-15

**Authors:** Sisi Wang, Gang Wang, Xuefei Liu, Jinshun Bi, Wenjun Xiao, Degui Wang, Mingqiang Liu, Changsong Gao, Ziqiang Xu, Zhen Wang, Yan Wu, Abuduwayiti Aierken

**Affiliations:** 1 School of Physics and Electronic Science, Guizhou Normal University, Guiyang 550025, China; 232100070252@gznu.edu.cn (S.W.);; 2 School of Integrated Circuit, Guizhou Normal University, Guiyang 550025, China

**Keywords:** smart hydrogels, temperature/pH dual-responsive, preparation methods, structural design strategies, biomedical applications

## Abstract

Temperature/pH dual-responsive hydrogels are a class of smart materials capable of undergoing reversible structural or functional changes in response to temperature and pH stimuli. Owing to their remarkable dual-stimuli-responsive characteristics, these hydrogels have demonstrated significant potential in various biomedical applications, including drug delivery, tissue engineering, and diagnostics technologies, making them a prominent research focus. Although considerable progress has been made in recent years, a systematic summary of the preparation methods, structural design strategies and complex biomedical applications of these materials remains conspicuously absent. Consequently, this review aims to comprehensively examine the latest advancements in this field. First, the primary preparation methods of temperature/pH dual-responsive hydrogels, including chemical crosslinking, physical crosslinking, and hybrid crosslinking, are introduced and compared. Subsequently, the main structural design strategies, including microsphere, core–shell and layered structures, and their corresponding fabrication processes are systematically elucidated. Finally, the recent progress of temperature/pH dual-responsive hydrogels in biomedical applications is discussed, including drug delivery, cancer therapy, biosensing and diagnosis, tissue engineering and regenerative medicine, as well as wound healing. Based on the current research progress, this review also outlines the major challenges in the development of temperature/pH dual-responsive hydrogels, and presents perspectives on future research directions.

## 1. Introduction

Smart materials, serving as the fundamental materials for interdisciplinary integration, demonstrate significant value across diverse applications owing to their dynamic responsiveness to environmental stimuli, including temperature, pH, light, magnetic fields and so on [1,2,3,4]. Among them, smart hydrogels possess high water content, soft elasticity, and superior biocompatibility, stemming from their three-dimensional crosslinked network structure [5,6]. Their ability to mimic the chemical and physical characteristics of human tissues renders them promising candidates for various biomedical applications, such as drug delivery, wound healing, and biosensing [7,8]. The dynamic and complex nature of the in vivo microenvironment necessitates a high degree of precision in material responsiveness [9,10]. However, conventional hydrogels are often inadequate to meet the regulatory requirements of dynamic physiological processes due to their restricted single-response mode or limited sensitivity, which makes multi-stimuli-responsive hydrogels a prominent research focus [11,12,13].

Among multi-stimuli-responsive hydrogels, temperature/pH dual-responsive hydrogels have attracted considerable attention due to their compatibility with key physiological characteristics, such as the inherent pH gradients and stable temperature regime of the human body [14,15]. While single-stimulus-responsive hydrogels (e.g., pH- or temperature-responsive only) can achieve elementary regulation, their utility is often limited by inadequate adaptability within complex physiological microenvironments [16,17,18]. For instance, the tumor microenvironment exhibits both near-body-temperature conditions (~37 °C) and acidic properties (pH 6.5–7.2), notably lower than those of normal tissues (pH 7.3–7.4) [19]. A hydrogel responsive to only one stimulus may trigger partial regulation, yet falls short of enabling precise release and targeted delivery [20]. Conversely, temperature/pH dual-responsive smart hydrogels adapt synergistically to these two critical parameters, thereby precisely matching the dynamic regulatory process in vivo [21]. The pH differential between healthy and pathological tissues (e.g., tumors and inflamed regions) offers a precise target for pH-based response, while the consistent internal temperature environment of the human body offers a reliable benchmark for thermal response [22,23]. This temperature/pH dual-responsive hydrogel synergy significantly expands the potential applications of such materials in biological contexts [24].

The application of temperature/pH dual-responsive smart hydrogels has demonstrated remarkable potential in the biomedical field in recent years [25]. These hydrogels enable precise and targeted drug delivery through the synergistic control of pH and temperature [26]. It can respond to the body temperature signals and acidic properties of the tumor microenvironment, extending its residence duration at the lesion site and enhancing the therapeutic impact [27]. Additionally, the hydrogels’ temperature/pH dual-responsive characteristic offers a novel approach for generating sensitive signal feedback and dynamic adaptation to physiological environment in the fields of tissue engineering and biosensing [28,29]. Despite these promising features, several significant challenges remain in practical applications. In terms of fabrication, physically crosslinked hydrogels often suffer from insufficient stability [30], whereas chemical crosslinked hydrogels may pose biocompatibility concerns [31]. Additionally, the complexity of the hybrid crosslinking process limits its reproducibility and scalability for large-scale synthesis [30]. In structural design, precisely controlling the accuracy of complex microstructures and achieving stable and reliable manufacturing remain a major challenge [31,32]. Practical application issues include uncontrolled drug release [33], limited tumor penetration [34], interference with sensing accuracy [35], and inadequate alignment between degradation rates and physiological processes [36,37]. Collectively, these challenges hinder the clinical development and widespread adoption of smart hydrogels.

**Figure 1:**
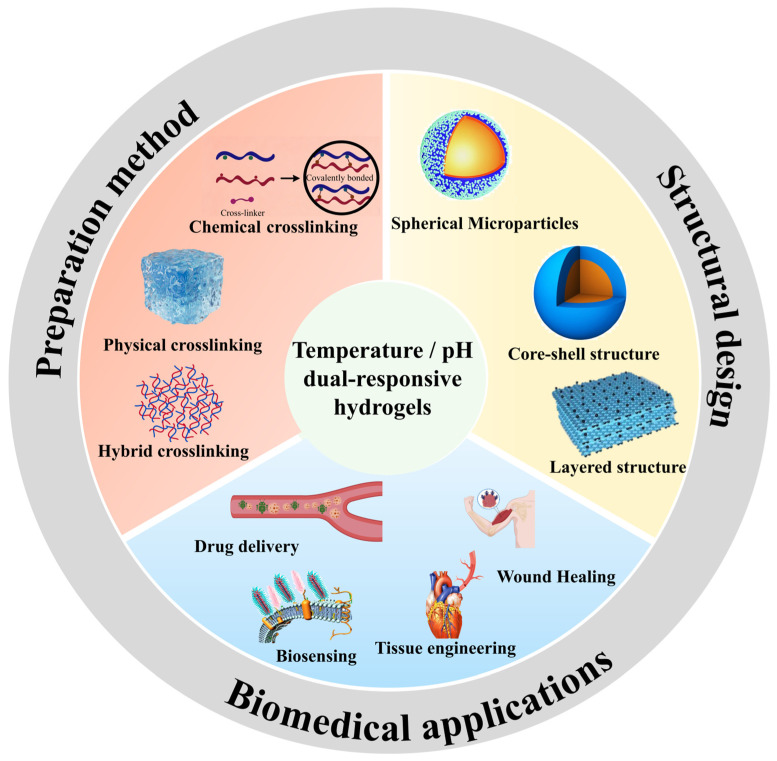
A schematic showing the outline of this review, including the preparation methods, structural design, and biomedical applications of temperature/pH dual-responsive smart hydrogels.

Several recent reviews have addressed various aspects of responsive hydrogels. However, a review specifically focusing on temperature/pH dual-responsive hydrogels—covering their preparation methods, structural design strategies, and biomedical applications—has been conspicuously lacking. For example, Guo et al. comprehensively elucidated the preparation strategies and response mechanisms of diverse stimulus-responsive nanocomposite hydrogels spanning temperature, pH, humidity, electrical, and light responses, yet did not provide a systematic comparison across different crosslinking types within a single material system [38]. Nunziata et al. systematically elaborated on the fabrication methodologies and design strategies of smart pH-responsive polymers for biomedical applications, covering nanoparticles, hydrogels, and emerging hybrid platforms, while focusing exclusively on pH sensitivity without addressing dual-responsive architectures [39]. Furthermore, Yadav et al. summarized the molecular engineering, dynamic crosslinking strategies, and therapeutic applications of thermo-responsive hydrogels, but their coverage is limited to temperature alone [40]. This review systematically integrates the preparation methods, structural design strategies, and biomedical applications of temperature/pH dual-responsive hydrogels. Furthermore, it highlights the main challenges currently and anticipates future research directions, aiming to provide a valuable reference for further studies and practical applications in this field. The overall structure of this review is depicted in Figure 1. This figure provides a visual roadmap of the review, illustrating how the preparation methods, structural design strategies, and biomedical applications of temperature/pH dual-responsive hydrogels are systematically connected.

## 2. Preparation Methods of Temperature/pH Dual-Responsive Hydrogels

Temperature/pH dual-responsive hydrogels can mimic the dynamic behavior of biological tissues by responding simultaneously to environmental temperature and pH stimuli, and thus exhibit broad potential for applications in advanced biomedical fields such as tissue engineering, flexible sensing, precise drug delivery, and actuation [41,42]. Regarding the responsive mechanisms of temperature/pH dual-responsive hydrogels, the temperature responsiveness typically arises from the lower critical solution temperature (LCST) behavior of polymers such as N-isopropylacrylamide (NIPAM) [43]. Below the LCST, polymer chains are hydrated through hydrogen bonding with water molecules, maintaining an extended and swollen state. Above the LCST, these hydrogen bonds are disrupted, and hydrophobic interactions become dominant, causing the polymer chains to collapse and the network to shrink. The pH responsiveness originates from ionizable functional groups (e.g., carboxyl –COOH) attached to the polymer backbone [44]. At low pH, carboxyl groups are protonated (–COOH, neutral), eliminating electrostatic repulsion and promoting network collapse. At high pH, they deprotonate to –COO^−^, increasing repulsion and swelling. For amino groups, the opposite behavior is observed. In dual-responsive systems, these two mechanisms can act synergistically, enabling precise spatiotemporal control in complex physiological environments such as tumors. The mechanical strength, stability, and response characteristics of temperature/pH dual-responsive hydrogels are predominantly governed by the crosslinking strategy employed in constructing their network. In this section, we briefly summarize three primary crosslinking approaches: chemical crosslinking, physical crosslinking, and hybrid crosslinking.

### 2.1. Chemical Crosslinking

**Figure 2:**
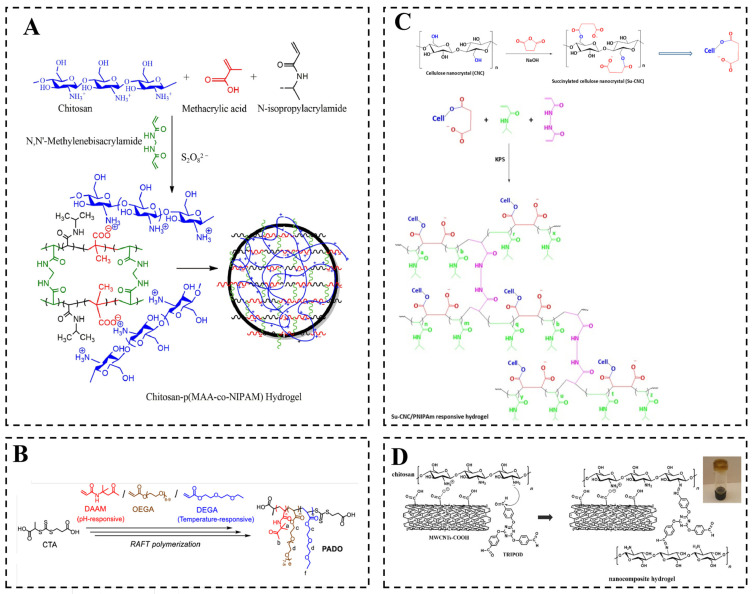
Hydrogel networks prepared by chemical crosslinking. (**A**) Chitosan-poly(methacrylic acid-co-N-isopropylacrylamide) (p(MAA-co-NIPAM)) hydrogel. (Reprinted from Ref. [54]. Copyright © 2018, Elsevier.) (**B**) Synthesis route of poly(acylhydrazone) (PADO) polymer via reversible addition–fragmentation chain transfer (RAFT) polymerization with pH-responsive acylhydrazone bonds. (Reprinted from Ref. [58]. Copyright 2022, Wiley-VCH GmbH.) (**C**) Structural design of Su-cellulose nanocrystal/poly(N-isopropylacrylamide) (CNC/PNIPAM) hybrid hydrogels. (Reprinted from Ref. [60]. Copyright © 2022, Elsevier.) (**D**) Temperature/pH dual-responsive chitosan hydrogel crosslinked by dynamic Schiff base reaction using 1,3,5-triazine-2,4,6-tribenzaldehyde (TRIPOD) crosslinker. (Reprinted from Ref. [61]. Copyright © 2017, Elsevier.).

Chemical crosslinking refers to the permanent interconnection of polymer chains through covalent bonds, forming a three-dimensional network that endows hydrogels with robust mechanical properties and high chemical stability [45,46,47,48,49,50]. Figure 2 shows a chemically crosslinked hydrogel that integrates temperature and pH responsiveness using covalent bonds and dynamic covalent bonds. On the basis of bond type and fabrication method, these strategies are categorized into four groups, as discussed below.

Conventional chemically crosslinked networks are predominantly based on static covalent bonds, such as ester, amide, and carbon–carbon linkages [51,52,53,54,55,56]. This structure provides high mechanical strength and excellent long-term stability. For instance, Xu et al. prepared Poly(N-isopropylacrylamide-co-acrylic acid) (p(NIPAM-co-AAc)) hydrogel by soap-free emulsion polymerization. The PNIPAM segment imparts temperature-responsive behavior characterized by the LCST, while the acrylic acid (AAc) carboxyl group achieves pH-responsive swelling by protonation. This structure allows tunable fractal features, thereby achieving dual stimuli-regulated drug release functions [53]. Similarly, Rasib et al. employed free-radical copolymerization of chitosan with NIPAM and methacrylic acid (MAA) to synthesize chitosan-p(MAA-co-NIPAM) hydrogel, in which the permanent network supported pH/temperature-dependent surface properties and controlled release (Figure 2A) [54]. Suryavanshi et al. developed NIPAM-co- 2,4′-diacryloyloxy benzophenone (DABP)-co-AAc hydrogel for 5-fluorouracil delivery, demonstrating how static covalent networks provide robust drug retention over extended periods [15]. Furthermore, Mohamed et al. constructed a network by reacting amino groups on chitosan with trimellitic anhydride isothiocyanate, introducing carboxyl and thiourea moieties that significantly enhanced antibacterial activity [55]. The primary functional advantage of static covalent crosslinking lies in its ability to confer long-term structural integrity, which is essential for achieving sustained and controlled release profiles [56]. However, a major limitation of this approach stems from the potential toxicity of residual crosslinkers or degradation products.

Dynamic covalent crosslinking relies on reversible bonds such as hydrazone, imine (Schiff base), boronic ester, and disulfide linkages [57,58,59,60,61]. This structural feature endows hydrogels with injectability and self-healing capability. For instance, Lin et al. employed reversible addition–fragmentation chain transfer (RAFT) polymerization to synthesize acylhydrazone-linked poly(acylhydrazone) polymer (PADO) hydrogels (Figure 2B) [58]. In this system, pH-responsive acylhydrazone bonds were formed between the hydrazide groups of the crosslinker adipic acid dihydrazide (ADH) and the ketone groups on the polymer side chains, enabling precise control over gelation, drug release, and degradation. Similarly, Emam et al. prepared an orally administrable interpenetrating network hydrogel using succinylated cellulose nanocrystals and PNIPAM, where dynamic boronic ester bonds provided dual-responsiveness (Figure 2C) [60]. Furthermore, Karimi et al. developed a chitosan-based hydrogel network through the dynamic Schiff base reaction between amino groups of chitosan and aldehyde groups of 1,3,5-triazine-2,4,6-tribenzaldehyde (TRIPOD). This reversible covalent structure provides self-healing ability under mild conditions and pH-triggered drug release (Figure 2D) [61]. The primary functional advantages of dynamic covalent crosslinking include the capability for injectable delivery and the potential for on-demand, stimulus-responsive drug release. However, its limitations lie in slower gelation kinetics and reduced mechanical robustness under cyclic loading.

Controlled polymerization and click chemistry enable the synthesis of well-defined polymer architectures with low dispersity and tunable crosslinking density. These methods also allow for the incorporation of multiple functional groups with high spatial precision. For instance, Rai et al. employed RAFT polymerization to synthesize bioconjugates comprising transparent poly(2-acrylamido-2-methylpropane sulfonic acid) (PAMPS) and PNIPAM segments, which improved enzyme activity under fluctuating pH and temperature conditions [62]. In a separate study, Luo et al. fabricated ultrathin hydrogel shells on magnetic particle surfaces via photo-initiated click chemistry, enabling ultrafast microenvironment sensing and crosslinking with nanoscale spatial precision [63]. Furthermore, Yang et al. utilized an azide–alkyne click reaction in combination with polylactic acid to construct a dual-crosslinked network exhibiting high robustness and durability [30]. The advantage of these approaches lies in the ability to engineer sophisticated multi-responsive systems with high reproducibility. However, this advantage is counterbalanced by the requirement for multi-step synthetic procedures, which can increase complexity and cost.

Surface-grafted polymer brush architectures feature polymer chains covalently tethered to a solid substrate, forming brush-like or crosslinked brush layers. Crosslinked polymer brushes have also been developed for temperature/pH dual-responsive applications [64,65,66,67,68,69,70,71]. Poly(2-N-morpholinoethyl methacrylate) (PMEMA) microgels exhibit distinct dual-responsiveness to both temperature and pH. Their thermo-responsive behavior originates from the LCST of PMEMA chains, whereas the pH responsiveness arises from the protonation/deprotonation of the pendant morpholino groups [65]. For instance, Demirci et al. prepared crosslinked PMEMA brush gels via in situ and surface-initiated RAFT polymerization. The grafted brush architecture provides a high surface area and rapid chain mobility, leading to fast temperature/pH responsiveness. These properties enable the reversible capture and release of target molecules over multiple cycles, rendering them suitable for bioseparation and sensing applications [66]. Similarly, Eroğlu et al. fabricated poly(acrylic acid-co-N-isopropylacrylamide) p(AAc-co-PNIPAM) copolymer brushes via surface-initiated photoinduced electron/energy transfer–reversible addition–fragmentation chain-transfer (SI-PET-RAFT) polymerization, demonstrating composition-dependent dual-responsive behavior to both temperature and pH [67]. Furthermore, Li et al. synthesized a temperature/pH dual-responsive hydrogel on a medical titanium alloy surface via atom transfer radical polymerization (ATRP), demonstrating good mechanical compatibility with human soft tissues and localized drug release [71]. These surface-grafted systems are particularly well-suited for implant coatings and biosensing applications. Their primary limitations, however, include the difficulty in scaling up the fabrication process for producing bulk hydrogel, as well as the complexity of surface functionalization.

### 2.2. Physical Crosslinking

**Figure 3:**
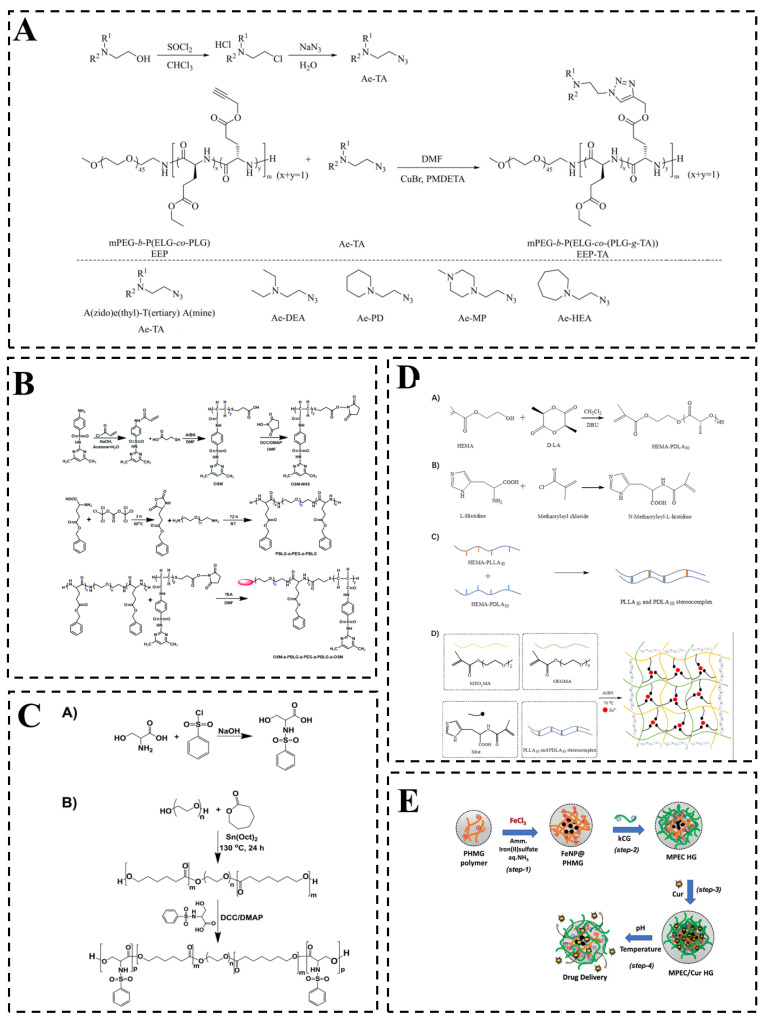
Hydrogel networks prepared by physical crosslinking. (**A**) Physically crosslinked temperature/pH dual-responsive hydrogels were formed by partial dehydration of monomethyl polyethylene glycol (mPEG) segments and secondary structure (α-helix to β-sheet) transformation. (Reprinted from Ref. [73]. Copyright 2023, Wiley-VCH GmbH.) (**B**) The oligo(sulfamethazine)-b-poly(γ-benzyl L-glutamate)-b-poly(ethylene glycol)-b-poly(γ-benzyl-L-glutamate)-b-oligo(sulfamethazine) (OSM-b-PBLG-b-PEG-b-PBLG-b-OSM) pentablock copolymer was synthesized by super-hydrophobic interaction. (Reprinted from Ref. [36]. Copyright 2018, Royal Society of Chemistry.) (**C**) Synthetic route for the preparation of A) serine sulfonamides (SSA) and B) pH/temperature dual-responsive pentablock copolymer by hydrophobic interaction and molecular chain entanglement. (Reprinted from Ref. [74] (**D**) Synthesis of A) hydroxyethyl methacrylate(HEMA)-PDLA30/PLLA30 macromonomers and B) mist monomer and C) stereocomplex of HEMA-PLLA30 and HEMA-PDLA30 and D) double-crosslinked hydrogel by physical double crosslinking of hydrogen bonds and metal coordination bonds. (Reprinted from Ref. [75]. Copyright © 2022, MDPI.). Copyright © 2019, Elsevier.) (**E**) Magnetic polyelectrolyte composite hydrogel (MPEC HG) was obtained by physical compounding through electrostatic interaction. (Reprinted from Ref. [76]. Copyright © 2023, Elsevier.)

In contrast to chemically crosslinked networks stabilized by permanent covalent bonds, temperature/pH dual-responsive hydrogels based on physical crosslinking form three-dimensional networks via dynamic and reversible non-covalent interactions, including hydrophobic association, hydrogen bonding, electrostatic interactions, and metal coordination and chain entanglement [72]. Figure 3 shows a physically crosslinked hydrogel that achieves temperature/pH dual-responsiveness using reversible non-covalent interactions.

Hydrophobic association is a common physical crosslinking mechanism, wherein nonpolar segments aggregate in aqueous environments to form transient crosslinks [73,74,77,78,79]. For example, Lin et al. developed a physically crosslinked polypeptide hydrogel in which network formation relies on hydrophobic interactions, the dehydration of poly(ethylene glycol) (PEG) segments, and secondary structure transition (α-helix to β-sheet) (Figure 3A) [73]. This dynamic non-covalent structure provides reversible gelation and pH responsiveness. These properties enable pH-dependent drug release with buffering capacity (pH 6.0–7.0), making the hydrogel suitable for localized cancer therapy. Turabee et al. designed an OSM-b-PBLG-b-PEG-b-PBLG-b-OSM pentablock copolymer that combines temperature-responsive poly(γ-benzyl-L-glutamate) (PBLG) segments with pH-responsive oligo(sulfamethazine) (OSM) segments via hydrophobic assembly, resulting in a hydrogel capable of efficiently loading and sustaining the release of cationic proteins (Figure 3B) [36]. Similarly, Nguyen et al. developed a biodegradable and bioresorbable temperature/pH dual-responsive pentablock copolymer with poly (ε-caprolactone)-b-poly (ethylene glycol)-b-poly (ε-caprolactone) (PCL-b-PEG-b-PCL) as a temperature-responsive triblock copolymer conjugated with two oligomer blocks of oligo (serine) (OS) as pH-responsive groups (Figure 3C) [74]. The structure enables the delivery of injectable sustained insulin. The functional advantage of hydrophobic association lies in its ability to confer injectability and drug release. Its main limitation, however, is poor long-term stability under physiological conditions, as the hydrophobic domains gradually dissociate.

Hydrogen bonding serves as a key physical crosslinking mechanism that links polymer chains into a network via interactions between polar groups (e.g., hydroxyl, carboxyl, or amide) on adjacent chains [75,80]. For example, Fallon et al. synthesized a temperature/pH dual-responsive hydrogel using itaconic acid (IA) as a pH-responsive monomer and N-vinylcaprolactam (NVCL) as a temperature-responsive monomer [80]. The system exhibited tunable dual-responsive swelling and drug release. By integrating multiple non-covalent mechanisms (e.g., coordination bonds, hydrogen bonds, and hydrophobic interactions) via rational molecular design, physically crosslinked hydrogels can be engineered to exhibit tailored responsiveness and enhanced mechanical properties [58,75,79,80]. A representative example is the work of Wu et al., who employed free-radical polymerization to copolymerize temperature-sensitive di(ethylene glycol) methyl ether methacrylate (MEO_2_MA) and oligo(ethylene glycol) methyl ether methacrylate (OEGMA), pH-responsive N-methacryloyl-L-histidine (Mist), and polylactide-based macromonomers (HEMA-PLLA_30_ and HEMA-PDLA_30_), resulting in a dual physically crosslinked network stabilized by hydrogen bonds and metal coordination bonds (Figure 3D) [75]. Such temperature/pH dual-responsive hydrogels effectively fabricated by these strategies show excellent potential in drug loading and controlled release. The reversibility and rapid response kinetics of hydrogen bonding render it particularly advantageous for applications in smart wound dressings and local drug delivery. However, hydrogels reliant solely on hydrogen-bonding generally suffer from insufficient mechanical strength, which limits their utility in load-bearing scenarios.

Electrostatic interactions can arise between oppositely charged polymer chains, as well as between charged polymers and their counterions. As an example, Santhamoorthy et al. developed a polyelectrolyte composite hydrogel where electrostatic interactions between positively charged polyhexamethylene guanidine (PHMG)-coated magnetic nanoparticles and negatively charged κ-carrageenan form the physical network. The structure provides magnetic responsiveness and pH-sensitive swelling, and these properties enable effective curcumin release in acidic environments (Figure 3E) [76]. The functional advantage of electrostatic crosslinking lies in its pH-dependent reversibility, which makes it particularly suitable for targeted drug delivery in acidic pathological tissues. However, this approach is limited by its susceptibility to variations in ionic strength and relatively weak mechanical properties.

Metal coordination and chain entanglement serve as additional physical crosslinking mechanisms. Coordination bonds between polymer ligands and metal ions provide stronger yet reversible crosslinks, while chain entanglement contributes to network connectivity. For instance, Liu et al. developed a physically crosslinked hydrogel with >3100% elongation and 0.5 MJ/m^3^ toughness using hydrophobic association and hydrogen bonding; this robust network enabled the effective treatment of infected wounds under dynamic mechanical stress [78]. Le et al. reported a poly(ethylene glycol)–poly(sulfamethazine ester urethane) (PEG-PSMEU) copolymer that formed a physically crosslinked network under physiological conditions through hydrogen bonding and hydrophobic interactions, enabling injectable adhesion for wound healing [79]. The functional advantage of combining metal coordination and chain entanglement is the ability to achieve high toughness and self-healing. However, this strategy presents notable limitations, primarily concerning the potential cytotoxicity of the constituent metal ions and the complexity of coordinating multiple crosslinking modes.

### 2.3. Hybrid Crosslinking

**Figure 4:**
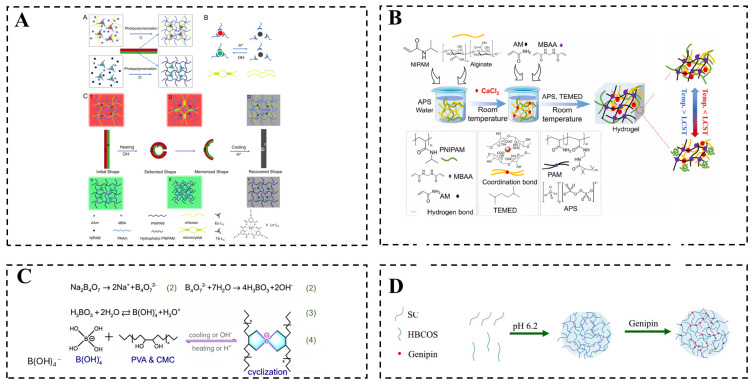
Hydrogel networks prepared by hybrid crosslinking. (**A**) A hybrid crosslinking network is formed by the integration of chemical crosslinking and physical crosslinking. A: Bilayer hydrogel was formed by two-step copolymerization. B: chitosan microcrystals dynamic reversible response under acid-base stimuli. C: Bending deformation of the bilayer hydrogel under alkaline condition and acidic condition with cooling. (Reprinted from Ref. [83]. Copyright 2022, American Chemical Society.) (**B**) A physical and chemical double crosslinked hydrogel prepared by combining covalent bonds and ionic bonds. (Reprinted from Ref. [84]. Copyright © 2025, Elsevier.) (**C**) Bilayer hydrogel with heterogeneous networks: physical crosslinking via poly(vinyl alcohol) (PVA) microcrystallites on one side, and both physical and chemical crosslinks on the other side. (Reprinted from Ref. [85]. Copyright 2023, American Chemical Society.) (**D**) After electrostatic interaction, the temperature-responsive composite nanoparticles (CNPs) with physical and chemical double crosslinking were obtained through dialysis and freeze-drying. (Reprinted from Ref. [86]. Copyright © 2024, Elsevier.).

Hybrid crosslinking combines two or more distinct crosslinking mechanisms within a single hydrogel system, most commonly by integrating covalent bonds with non-covalent interactions or by incorporating nanomaterials as multifunctional crosslinkers [52,81]. Figure 4 shows a hybrid crosslinked strategy combining covalent and non-covalent bonds or incorporating nanomaterials.

Chemical–physical dual networks feature both permanent covalent bonds and reversible non-covalent interactions [82]. The covalent framework within this dual-crosslinked architecture imparts high mechanical strength. For instance, Li et al. fabricated a bilayer hydrogel using lanthanide complexes as multifunctional nodes that enabled covalent-coordination crosslinking, complemented by physical crosslinking from chitosan microcrystal (Figure 4A) [83]. This hybrid network exhibited enhanced mechanical strength, shape memory, and luminescence switching under temperature/pH stimuli. In another study, Liu et al. successfully synthesized a dual-crosslinked hydrogel via a simplified one-step free-radical polymerization that concurrently established *N*,*N*′-methylenebisacrylamide (MBA)-mediated covalent crosslinks and Ca^2+^-induced ionic crosslinks (Figure 4B) [84]. The resulting interwoven hybrid structure exhibits synergistic mechanical properties, enabling continuous drug release while maintaining excellent mechanical strength and biocompatibility. Furthermore, Jiang et al. used polyvinyl alcohol (PVA) as the polymer matrix, and carboxymethyl cellulose (CMC) as the structure and pH-responsive enhancement component. The bilayer strategy was employed to construct heterogeneous networks in the hydrogels: the physical crosslinking of PVA chains through the formation of PVA microcrystallites on one side and the coexistence of the aforementioned physical crosslinks and chemical crosslinks between PVA and CMC chains via B–O bonding on the other side (Figure 4C) [85]. In their work, the borate ester bonds played a dual role as both structural crosslinks and pH-responsive units. These properties enable multi-stimuli-triggered actuation. The functional advantage of chemical–physical dual networks is their exceptional comprehensive performance, including durability, injectability, and spatiotemporal control. Their limitations include complex fabrication processes, batch-to-batch variability, and challenges in achieving network homogeneity.

Nanoparticle-reinforced networks represent another pivotal strategy for simultaneously reinforcing and functionalizing hydrogels through the incorporation of nanomaterials into hybrid crosslinked networks [87,88]. Nanoparticles can not only serve as physical crosslinking sites within the polymer matrix but also engage in specific chemical interactions, collectively establishing a multiphase hybrid enhancement system [89]. For instance, Narendra et al. intercalated human growth hormone into layered double hydroxide nanoparticles and dispersed the complex into a cationic temperature/pH dual-responsive injectable hydrogel [88]. The nano-biohybrid structure enabled sustained hormone delivery with nanostructure-controlled release kinetics. Lu et al. introduced amine-functionalized iron oxide nanoparticles into a gelatin/polyglucuronic acid network [90]. Electrostatic interactions initially enable the physical integration of nanoparticles with the polymer, while the surface amino groups subsequently form dynamic imine bonds with aldehyde groups in the matrix, yielding a nanocomposite double-network hydrogel that exhibits magnetic responsiveness, enhanced mechanical properties, and multi-stimuli sensitivity. Similarly, Srikhao et al. incorporated silver nanoparticles, synthesized via a green method, into a tannic-acid-crosslinked network of polyvinyl alcohol and carboxymethyl starch [91]. The physical association between the nanoparticles and the polymer matrix further stabilizes the network, endowing the hydrogel with combined pH-responsive drug release and photothermal antibacterial capabilities. Furthermore, Chen et al. fabricated composite nanoparticles from hydroxybutyl chitosan oligosaccharide and sodium caseinate through a two-step process involving electrostatic complexation followed by genipin crosslinking (Figure 4D) [86]; the obtained nanoparticles exhibit structural stability and drug release profiles that can be synergistically modulated by temperature and pH variations. The primary functional advantage of this strategy lies in its capacity to integrate multiple functionalities into a single responsive platform. However, it is accompanied by several limitations, including potential nanoparticle toxicity, difficulty in achieving uniform dispersion, and increased complexity in both synthesis and characterization processes.

Table 1 systematically compares these methods in terms of their core bonding types, key advantages, and limitations. When considering specific biomedical applications, the choice of crosslinking strategy should be guided by the performance requirements of the intended use. For drug delivery applications requiring sustained release over days to weeks, chemically crosslinked hydrogels offer excellent structural stability and tunable degradation kinetics. However, the potential toxicity of residual crosslinkers raises major safety concerns for short-term use. In contrast, physically crosslinked hydrogels provide superior biocompatibility and injectability, making them particularly suitable for local minimally invasive delivery, yet their poor long-term stability limits their applicability in prolonged release scenarios. Hybrid crosslinked hydrogels combine the mechanical robustness of covalent networks with the reversibility of non-covalent interactions, which is especially advantageous for cancer therapy, where the tumor microenvironment demands precise spatiotemporal control. However, their fabrication is complex, batch-to-batch reproducibility is poor, and scalability remains challenging. For tissue engineering scaffolds that require mechanical integrity over months, chemical or hybrid crosslinked hydrogels are preferred, whereas for wound dressings that prioritize rapid response and biocompatibility, physical crosslinked hydrogels may be more appropriate. In summary, the choice of crosslinking strategy should be dictated by the specific application context.

## 3. Structural Design Strategies and Typical Fabrication Methods

Temperature/pH dual-responsive hydrogels represent a prominent class of smart materials in biomedicine, characterized by their ability to undergo controlled structural and functional changes in response to specific physiological or external stimuli [44,93]. By tailoring their chemical composition and network architecture, the responsiveness of these hydrogels can be precisely engineered to match complex biological microenvironments. Ultimately, the performance of these hydrogels, including response kinetics, functionality, and practical applicability, is fundamentally governed by the synthesis strategy and the resulting structural morphology. This section provides a systematic review of the prevailing structural design strategies, including microsphere structure, core–shell structure and layered structure, and briefly introduces their corresponding typical fabrication methods.

### 3.1. Microsphere Structure

Microspheres, as a fundamental spherical architecture, are characterized by high uniformity and morphological homogeneity [94]. These hydrogel particles typically range in size from nanometers to several micrometers in diameter. A key technique for producing monodisperse microspheres is precise droplet generation using microfluidic techniques. For example, Haney et al. employed a polydimethylsiloxane (PDMS)-based microfluidic system to fabricate highly uniform Janus droplets [95], and subsequent photopolymerization and hydrolysis yielded Janus microgels with a distinctive biphasic hemispherical structure, in which the hydrophilic and hydrophobic domains can be independently regulated by pH and temperature. To achieve composite microcapsules with enhanced mechanical properties and multimodal responsiveness, Cao et al. utilized centrifugal microfluidic technology to prepare nanocomposite hydrogel microcapsules with an average diameter of approximately 298.7 μm [31]. This design integrates responsiveness to temperature, pH, magnetic field, and near-infrared light. Despite the challenges associated with large-scale production, the resulting architecture exhibits high stability and effectively prevents oil phase contamination.

Large-scale preparation of microspheres is commonly achieved through various emulsion polymerization techniques. For instance, Wan et al. employed inverse microemulsion polymerization to synthesize PNIPAM/itaconic acid nanogels with an average particle size of 49 nm in a cyclohexane oil phase [96]; these nanogels exhibited broad temperature and pH responsiveness, but challenges related to residual organic solvents and surfactants remained to be addressed. In a separate study, Lai et al. fabricated temperature/pH dual-responsive microspheres with an average diameter of approximately 6.5 μm and a semi-interpenetrating network structure through first generated homogeneous emulsion droplets via premixed membrane emulsification and followed by one-step suspension polymerization [97]. The resulting microspheres exhibited stable and independent response behavior suitable for long-term storage, and their properties were highly sensitive to process parameters such as transmembrane pressure. Moreover, surfactant-free precipitation polymerization offers a route that avoids the complexity associated with emulsifier addition. Using this method, Xiao et al. directly prepared highly monodisperse microgels with a diameter of approximately 592 nm [98]. Although precise control over monomer ratio and reaction conditions remains challenging, the obtained microgels possess a clean surface that facilitates subsequent functionalization.

Specialized preparation strategies are often employed to meet specific material property or functional requirements. For example, Zhang et al. fabricated hollow porous ZnO microspheres with hierarchical pore structures via a combination of solvothermal synthesis and subsequent heat treatment [99]. These microspheres exhibit high specific surface area and pH-responsive behavior, making them promising drug carriers. However, synthesis involves multiple steps and is relatively energy-consuming. In a distinct approach, Isapour et al. demonstrated the assembly of colloidal nanogels as building blocks within emulsion-templated microspheres via photopolymerization, leading to the formation of internal photonic crystal structures [94]. This strategy enabled the fabrication of sensing microspheres capable of rapidly modulating their structural color in response to changes in temperature and pH.

### 3.2. Core–Shell Structure

A core–shell structure typically consists of an inner core and an outer shell. This spatial compartmentalization allows distinct responsive components to be localized in separate domains, enabling precise and coordinated control over stimuli-responsive behaviors. Among the most mature and versatile techniques for constructing such architectures, the layer-by-layer (LbL) assembly method holds an important position. For example, Theodorakis et al. successfully fabricated single- or double-layer hybrid core–shell nanoparticles by alternately depositing the graft copolymer NaALG-g-P(NIPAM-co-NtBAM) and poly(allylamine hydrochloride) (PAH) onto mesoporous silica nanoparticles (MSNs) [32]. Although this approach offers precise control over drug release kinetics through the number of shell layers, it involves multiple centrifugal washing steps, which are labor-intensive and may lead to drug loss. Similarly, Hammad et al. utilized layer-by-layer (LbL) assembly to alternately coat poly(allylamine) (PAH) and poly(acrylic acid) (PAAc) onto dual magnetic nanoparticles, forming a polymer shell whose thickness could be adjusted by the number of layers, which in turn precisely regulated drug release rates [100]. However, the coating process reduces the effective mass fraction of the magnetic core and adds complexity to the overall fabrication.

In addition to LbL assembly, several alternative strategies have also been developed for constructing core–shell architectures. For instance, Zhang et al. adopted a static template polymerization approach, using popcorn pollen as a natural core and performing stepwise polymerization to grow a temperature-responsive shell around it, thereby obtaining a rough, porous core–shell hydrogel [101]. This method is straightforward and well-suited for incorporating natural materials, but achieving uniform interfacial contact between the core and shell remains challenging. In a conceptually related design, Lee et al. employed two-photon polymerization-based three-dimensional (3D) printing to fabricate a spherical p(NIPAM-co-AAc) structure, which functionally embodies a core–shell design principle, although its primary purpose is to serve as a microrobot [102]. Additionally, Najafipour et al. utilized ultrasound-assisted emulsion polymerization to fabricate core–shell magnetic nanocomposite particles for cancer combination therapy, in which Fe_3_O_4_ nanoparticles are used as the magnetic core and responsive polymers as the outer shell [103].

### 3.3. Layered Structure

Bilayer structures of hydrogels are constructed by integrating two distinct gel layers with unique stimuli-responsive properties through physical or chemical methods. This architectural design exploits the complementary mechanical behaviors and synergistic responses of the constituent layers to achieve precise stimulus-response matching and advanced functional integration. Among the strategies for fabricating such systems, LbL self-assembly has emerged as a widely adopted technique due to its high controllability over interlayer architecture, ease of functional component incorporation, and operational simplicity. For instance, Gundogdu et al. alternately deposited a temperature-responsive polymer (PiPOX) and tannic acid (TA) onto alginate (ALG) hydrogels via hydrogen-bond-directed LbL assembly, endowing the natural hydrogel with a temperature/pH dual-responsive surface coating that enabled pH- and temperature-synergistic drug release for infected wound environments [104]. Similarly, Theodorakis et al. utilized LbL self-assembly to construct a polyelectrolyte shell on mesoporous silica, allowing the precise regulation of drug release kinetics [32]. In another approach, Zavgorodnya et al. employed LbL self-assembly to alternately deposit sacrificial layers and temperature-sensitive nanogels, and subsequently removed the sacrificial layers to obtain a multilayer film with a three-dimensionally interconnected network. The film exhibited synergistic drug release behavior at skin-relevant temperature and pH [105]. In addition to LbL self-assembly, other polymerization-based methods have also been developed to manufacture bilayer actuators. Lin et al., for example, fabricated a PNIPAM/sodium polyacrylate (PSA) Janus hydrogel through a straightforward stacking-polymerization method [106]. This bilayer structure undergoes reversible bending driven by asymmetric swelling in response to solvent and temperature changes, enabling it to grasp and release objects in hazardous environments. Jiang et al. produced a temperature/pH dual-responsive hydrogel actuator capable of bidirectional bending via a two-step UV polymerization technique [107]. The resulting structure achieves large bending angles with rapid response, showing promising potential in soft robotics, biomimetic structures, and micro-manipulation systems. Additionally, Leow et al. prepared heterogeneous bilayer hydrogels with strong interfacial fusion by pre-assembling different monomers and followed by one-step photopolymerization based on halogen-bond-mediated solid-phase free-radical polymerization, thus realizing complex and reversible shape transformations [108].

The functionality of bilayer hydrogel structures can be substantially enhanced by introducing an intermediate transition layer. This additional layer not only improves interfacial compatibility but also enables more sophisticated synergistic responses. For example, Chen et al. fabricated a sandwich-structured actuator by in situ polymerization of a temperature/near-infrared responsive PNIPAM layer and a pH-responsive PAAc layer on the opposite surfaces of an anisotropic natural bamboo-chip skeleton [109]. The resulting trilayer architecture was capable of executing complex shape transformations, such as curling into rings and spirals. In another approach, Zhou et al. utilized mask stereolithography (mSLA)-based 4D printing to sequentially construct a three-layer system consisting of a pH-responsive hydrogel, an adhesive intermediate layer, and a temperature-responsive shape-memory polymer [110]. By strategically incorporating the adhesive interlayer, the incompatibility between hydrophilic and hydrophobic materials was resolved, enabling precise spatiotemporal control over actuation triggered synergistically by pH and near-infrared light. This design effectively converts two independent stimulus-response signals into a unified mechanical motion by exploiting the distinct material properties and their structural integration of each layer.

A more integrated design strategy achieves a functional bilayer within the same temperature/pH dual-responsive material by spatially regulating differentiated crosslinking density during the fabrication process. For instance, Wang et al. employed femtosecond laser direct writing (FsLDW) to fabricate asymmetric micro-arm structures from a uniform temperature/pH dual-responsive hydrogel, in which the crosslinking density was spatially graded [111]. This structural asymmetry induces differential swelling and shrinkage behaviors under identical environmental stimuli. As a result, the microactuators can independently respond to a single stimulus or produce coordinated deformation when both stimuli coexist, thereby enabling operations such as capturing and releasing 10 μm polystyrene microspheres. Crucially, even with a chemically homogeneous composition, the programmed asymmetry in crosslinking drives directional bending and allows the response amplitudes to temperature and pH to be modulated separately. This allows the realization of sophisticated cooperative actuation from a single material platform.

Table 2 presents a comparative analysis of the underlying principles, functional advantages, key limitations, and representative applications associated with each design paradigm. The three structural design strategies described above each offer distinct advantages for different biomedical applications. Microsphere structures, characterized by their high specific surface area and rapid mass transfer, are best suited for injectable drug delivery and local immunomodulation, where quick release and efficient cellular uptake are desired. However, their small size limits the drug loading capacity and makes the scalable production challenging. Core–shell structures excel in sequential or cascade release applications, particularly in cancer therapy requiring on-demand drug release triggered by the tumor microenvironment. In such designs, the shell acts as both a protective and a gate-keeping layer. Nevertheless, the multi-step fabrication process and the difficulty in achieving uniform shell thickness remain major drawbacks. Layered structures are uniquely capable of generating bending deformation, rendering them ideal for soft robotics, biomimetic actuators, and smart wound dressings that require mechanical movement. Their primary limitation lies in poor interfacial stability between adjacent layers and high demands on fabrication precision. In summary, microsphere structures are preferred for rapid-release injectable formulations, core–shell structures for programmed multi-stage release, and layered structures for actuation and shape-morphing applications. The choice of architecture should be dictated by the functional demands of the target biomedical problem.

## 4. Biomedical Applications of Temperature/pH Dual-Responsive Hydrogels

**Figure 5:**
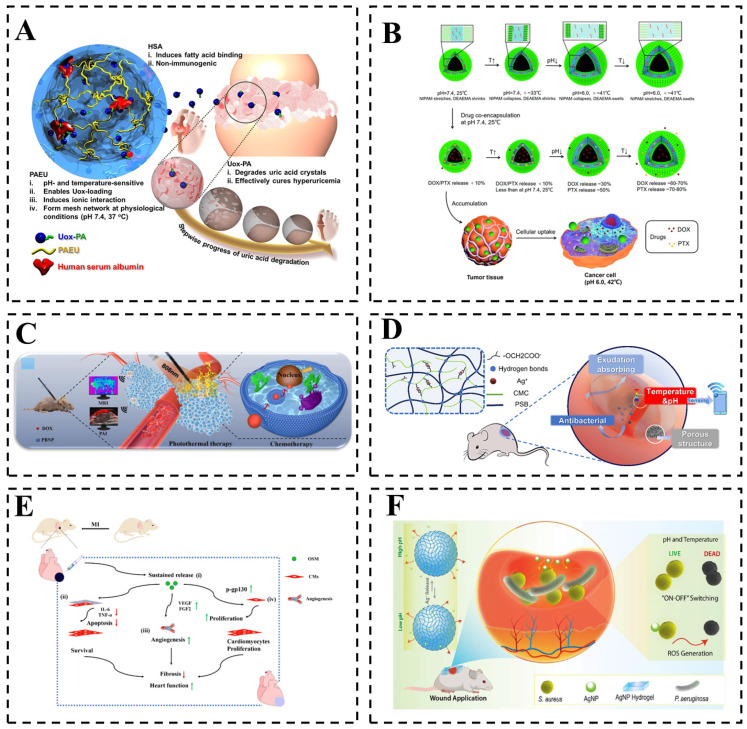
The specific application of biomedicine. (**A**) The stable loading and sustained release of biopharmaceutical uricase (Uox) were achieved by pH and temperature dual-responsiveness. (Reprinted from Ref. [117]. Copyright © 2017, Elsevier.) (**B**) Doxorubicin (DOX) and paclitaxel (PTX) encapsulated in temperature/pH dual-responsive polymersomes for killing cancer cells. (Reprinted from Ref. [118]. Copyright © 2018, Elsevier.) (**C**) Using tumor chemo-photothermal combination therapy, temperature/pH dual-responsive hydrogel for human breast cancer cell line (MCF-7) tumor treatment. (Reprinted from Ref. [33]. Copyright © 2022, Elsevier.) (**D**) Hydrogel skin sensor integrating real-time monitoring, antibacterial and healing promotion. (Reprinted from Ref. [119]. Copyright © 2022, Elsevier.) (**E**) Oncostatin M (OSM)-loaded temperature/pH dual-responsive hydrogels release OSM to promote angiogenesis and inhibit myocardial fibrosis. (Reprinted from Ref. [120]. Copyright 2022, Royal Society of Chemistry.) (**F**) Ultra-small silver nanoparticles loaded on responsive hydrogels for wound healing at the infected site of scald. (Reprinted from Ref. [121]. Copyright © 2025, Elsevier.

Temperature/pH dual-responsive hydrogels have become the most representative biomaterial for biomedical applications due to their biocompatibility and similarity with biological soft tissue [112,113]. Their high biosafety upon contact with biological tissues, cells, or bodily fluids establishes a foundation for their application in both in vitro and in vivo biomedical settings [114,115]. These hydrogels show considerable promise in fields such as tissue engineering, controlled drug release, biosensors, and smart wound dressings [93,116]. This section will methodically review the specific application contexts of temperature/pH dual-responsive hydrogels in biomedicine, as well as the challenges associated with their practical implementation. Figure 5 summarizes the biomedical application of temperature/pH dual-responsive hydrogels, including drug delivery, cancer therapy, wound healing, and tissue engineering.

### 4.1. Drug Delivery

One of the goals in the biomedical field is to improve therapeutic efficacy and minimize side effects through precise and efficient drug delivery. Owing to their responsiveness to in vivo microenvironmental signals, temperature/pH dual-responsive hydrogels enable precise control over the timing, rate, and location of drug release in response to environmental stimuli [53,59,102,122,123,124]. This capability overcomes key limitations of conventional drug delivery systems, namely poor targeting specificity and low release accuracy. Gil et al. developed a hybrid injectable hydrogel that utilized its dual-responsiveness to temperature and pH to achieve steady loading and prolonged release of the enzyme uricase (Uox), with approximately 80% of the therapeutic protein retained within the hydrogel network after 5 days of dialysis against PBS (pH 7.4, 37 °C), thereby extending the in vivo half-life from 4.3 h to 25.8 h in a murine model of hyperuricemia and enhancing the therapeutic outcome (Figure 5A) [117]. Using ibuprofen (Ibu) and 5-fluorouracil (5-Fu) as model drugs, Mohan et al. fabricated a PNIPAM-co-PGMA-Mela temperature/pH dual-responsive hydrogel and evaluated its drug release performance using Ibu and 5-Fu as model drugs. Under conditions of pH 4.0 and 45 °C, the hydrogel achieved nearly 100% cumulative release for both drugs, demonstrating excellent dual-responsive controlled release capability [125]. The controlled synthesis and stimulus-responsive properties of such hydrogels can be harnessed to achieve a synergistic effect combining antibacterial action with targeted drug release in the treatment of inflammatory lesions [126]. Nawaz et al. created amorphous hydrogels with a 3D network and nanocage structure for the controlled release of loxoprofen (LXM) [127]. This system presents a novel strategy for the localized and long-term controlled delivery of drugs, showing particular promise for managing conditions such as arthritis and postoperative inflammation. The application of temperature/pH dual-responsive hydrogels also extends to the delivery of protein-based therapeutics [36,58]. Komatsu et al. developed a biodegradable hydrogel capable of responding to multiple stimuli [128]. The temperature-responsive component contributes to protein loading and subsequent release at specific pH values (5.4 or 7.4), allowing for release profiles to be tailored to the acidic tumor microenvironment.

Owing to its high patient compliance and ease of use, oral administration remains the preferred route for clinical drug delivery. Temperature/pH dual-responsive hydrogels can be precisely engineered to respond to body temperature and the gastrointestinal pH gradient, offering a novel strategy for the stable delivery and targeted release of oral therapeutics. For instance, Cheng et al. developed a temperature/pH dual-responsive hydrogel for oral delivery of calcitonin. The encapsulation efficiency exceeded 96%, and in simulated intestinal fluid (pH 6.8), the drug release reached approximately 100% within 24 h. The hydrogel also exhibited good biocompatibility, with 293T cell viability of 79–84% after 3 d of culture [129]. The responsive properties of temperature/pH dual-responsive hydrogels have been successfully leveraged for the loading of protein-based drugs in studies focusing on site-specific oral delivery to the small intestine and colon [130]. By exploiting the environmental sensitivity of pH-responsive materials, drug release can be controlled to ensure effective intestinal absorption while preventing premature release and degradation in the upper gastrointestinal tract, thereby enhancing therapeutic efficacy [131]. Li et al. synthesized a hydrogel copolymer for the oral delivery of 5-Fu [132]. The cumulative drug release rate in simulated intestinal fluid (SIF, pH 7.4) reached 49.23%, substantially higher than the 9.27% observed in simulated gastric fluid (SGF, pH 1.2). Combined with a cell viability rate exceeding 90%, these results indicate excellent potential for targeted delivery along with high biocompatibility. Ferdous et al. introduced decanoyl glycol chitosan as a novel pH-responsive injectable thermogel, exhibiting a multiphase sol–gel–sol transition tunable by both temperature and pH [133].

Temperature/pH dual-responsive hydrogels exploit their dual sensing capabilities to precisely respond to and modulate the tumor microenvironment (TME) [20,76], thereby addressing the limitations of conventional chemotherapeutics, such as high systemic toxicity and poor tumor targeting. This unique characteristic enables spatiotemporally controlled drug delivery, encompassing targeted accumulation [31], local retention, and controlled release [30,86], which collectively enhances the precision and safety of cancer treatment. Anticancer drugs can be incorporated into the responsive hydrogel network to promote precise release triggered by specific pathological conditions within the TME, such as acidity and mild hyperthermia [134].

The clinical efficacy of doxorubicin (DOX), a broad-spectrum chemotherapeutic agent, is often limited by drug resistance and off-target toxicity. For example, Farjadian et al. synthesized DOX-conjugated nanogels (PVC-DOX) via RAFT polymerization using monomers including NVCL, lysine, and polyethylene glycol diacrylate (PEGDA) [135]. In a simulated TME (pH 5.0, 40 °C), approximately 80% of the encapsulated DOX was released over 72 h, resulting in significant cytotoxicity against MCF-7 breast cancer cells. The nanogels were effectively internalized by the cancer cells and distributed in the cytoplasm and nucleus, which is conducive to efficient drug release and targeted therapy. Similarly, Aminoleslami et al. synthesized a p(VCL-co-AAc) nanogel loaded with DOX. The nanogel achieved a drug loading efficiency of 83% and exhibited accelerated DOX release in a simulated tumor microenvironment (pH 5.4, 40 °C) compared to physiological conditions. The nanogel showed enhanced cytotoxicity against MCF-7 breast cancer cells, with a lower IC50 than free DOX [136]. Furthermore, Luo et al. developed a temperature/pH dual-responsive hydrogel as a carrier of 5-fluorouracil for triple-negative breast cancer treatment [137].

Curcumin, a natural polyphenolic compound, has been demonstrated to inhibit tumor cell proliferation, induce apoptosis, and suppress tumor angiogenesis and metastasis in both in vitro and in vivo models [20]. When incorporated into temperature/pH dual-responsive hydrogels, curcumin exhibits excellent controlled release profiles and biocompatibility, rendering this system a promising strategy for cancer therapy [118]. Similarly, paclitaxel (PTX), another natural anticancer agent, has been integrated into such responsive systems. For instance, Kang et al. developed an in situ-forming gelatin-OSM hydrogel for PTX delivery. In the tumor microenvironment (pH 6.5), the hydrogel released 63% of PTX over 9 d and significantly inhibited U87MG glioma cell viability (approximately 35% survival at a 1:100 ratio). In an orthotopic GBM model, the bioluminescence intensity at 9 d was only 287% of the initial value, effectively preventing tumor recurrence [41]. This system thus serves as a novel platform for local therapy of residual brain malignancies after resection. In another study, Zhou et al. fabricated temperature/pH dual-responsive polymersomes capable of co-encapsulating the hydrophilic drug DOX and the hydrophobic PTX, exhibiting synergistic cytotoxicity against MCF-7 and HeLa cells [134]. In a simulated tumor microenvironment (pH 6.0, 45 °C), the release of DOX reached 60–70% and PTX reached 70–80% within 4 d. These polymersomes represent a versatile co-delivery platform for combinatorial chemotherapy (Figure 5B). To mitigate the severe side effects of conventional chemotherapeutics such as DOX, a smart nanogel based on chitosan and p(NIPAM-co-AAc) was developed [119]. This hydrogel leverages pH-responsive behavior for on-demand drug release within the acidic tumor microenvironment, while its temperature-responsive properties improve systemic stability and enhance tumor-targeted accumulation. In mouse models bearing Ehrlich ascites carcinoma, the temperature/pH dual-responsive hydrogel exhibited enhanced antitumor efficacy together with markedly reduced liver, kidney, and DNA toxicity. Collectively, these findings underscore the critical role of intelligent stimulus-responsive carriers in realizing potent cancer therapy while minimizing damage to healthy tissues.

The integration of magnetothermal therapy with chemotherapy significantly enhances antitumor efficacy compared with either treatment alone, while the temperature/pH dual-responsiveness of the delivery system enables spatiotemporally controlled drug release. Najafipour et al. fabricated magnetic nanocomposite particles (MNCPs) composed of NIPAM, 2-(diethylamino)ethyl methacrylate (DEAEMA), Fe_3_O_4_ nanoparticles, and 3-(trimethoxysilyl)propyl methacrylate (TMSPMA) [103]. Under simulated tumor microenvironment conditions (pH 5.5, 42 °C), the MNCPs achieved 87% cumulative drug release over 42 h. The combined application of MNCPs and magnetic hyperthermia reduced the viability of MCF-7 cells to 28%, corresponding to an additional 17% reduction compared with free methotrexate (MTX). These results demonstrate a pronounced synergistic effect between magnetic hyperthermia and targeted drug delivery. Furthermore, the functionality of temperature/pH dual-responsive systems can be expanded by integrating them with other external stimuli. Chen et al. developed an injectable hydrogel responsive to temperature, pH, and near-infrared (NIR) light, thereby combining photothermal therapy with chemotherapy (Figure 5C) [33]. This combinatorial approach exhibited remarkable therapeutic efficacy in MCF-7-tumor-bearing mouse models. Advancing this concept further, Li et al. engineered a suite of cluster-responsive photonic nanorobots (RPNRs)—including pH-responsive, temperature-responsive, and glucose-responsive variants—using Fe_3_O_4_ nanoparticles as multifunctional cores and monomers such as AAc, 2-hydroxyethyl acrylate (HEA), and NIPAM [34]. These RPNRs integrate magnetic cluster motion, responsive structural color, and photothermal conversion. This platform established, for the first time, a closed-loop system for cancer therapy that enables a transition from active localization to signal mapping and, ultimately, to precise treatment.

To provide a quantitative comparison of the drug delivery performance among representative temperature/pH dual-responsive hydrogels, key parameters including drug loading efficiency, release kinetics, and anticancer efficacy are summarized in Table 3.

### 4.2. Biosensing and Diagnostics

Temperature/pH dual-responsive hydrogels can precisely sense changes in these parameters and transduce environmental cues into measurable chemical or physical signals [35]. Jiang et al. synthesized a bilayer temperature/pH dual-responsive hydrogel for use in flexible sensors capable of monitoring temperature variations, including those induced by breathing and proximity to hot and cold objects [85]. The resulting flexible sensors, which exhibit high tensile strength, sensitivity, rapid response, and fatigue resistance, can also be employed to monitor human motion [10,106]. Most notably, the unique properties of this bilayer hydrogel render it a promising candidate for medical diagnostics. For instance, Long et al. fabricated a zwitterionic cellulose-based hydrogel skin sensor with temperature/pH dual-responsiveness, which significantly accelerated wound healing in a mouse skin defect model [119]. Its integrated capabilities of antibacterial activity, healing promotion, and real-time monitoring provide a novel strategy for managing and monitoring infected wounds, even in non-clinical settings (Figure 5D). Furthermore, Shen et al. developed a pH-regulated and temperature-responsive reprogrammable hydrogel [29]. Under alkaline conditions, the hydrogel exists as a transparent, soft gel with a 3D network structure, whereas under acidic conditions, it transitions into an opaque, hard gel with a dense network. When employed as an adhesive electrode, its low skin-contact impedance (4.1 × 104 Ω) enables the clear monitoring of electromyography (EMG) and electrocardiogram (ECG) signals. Beyond these examples, Conejo-Cuevas et al. developed a self-healing, piezoresistive, and temperature-responsive chitosan/polyacrylic acid dynamic hydrogel, which exhibited a gauge factor of 0.23 under compressive strain and maintained sensing stability over 1000 cycles, demonstrating potential for wearable motion monitoring [35]. Additionally, temperature/pH dual-responsive hydrogels have been explored for biomarker detection. For instance, temperature/pH dual-responsive microgels functionalized with fluorescent dyes enabled photoacoustic imaging and temperature mapping in simulated physiological environments [98]. The combination of responsive swelling/deswelling with embedded reporting units (e.g., fluorophores and nanoparticles) allows these hydrogels to transduce environmental changes into optical or electrical signals with high spatiotemporal resolution. Owing to their excellent sensitivity and specific responsiveness, temperature/pH dual-responsive hydrogels show great promise in areas including biomarker monitoring, early disease detection, and dynamic in vivo microenvironment assessment, thereby offering sophisticated material solutions for advanced biosensing and diagnostics.

### 4.3. Tissue Engineering and Regenerative Medicine

The structural and functional reconstruction of damaged tissues can be enhanced by developing repair systems that mimic the dynamic in vivo microenvironment. Su et al. engineered heterogeneous porous scaffolds with multi-branched blood vessels (HPS-MFVs) by integrating 3D printing with stimuli-responsive hydrogels [138]. They successfully co-cultured hepatocytes (L02) and human umbilical vein endothelial cells (HUVECs) within these scaffolds to construct a bioengineered liver model. This approach provides a viable strategy for constructing complex vascular networks, thereby facilitating organ repair and regeneration in tissue engineering and regenerative medicine. Yu et al. developed an injectable, self-healing hydrogel with dual physical crosslinking. The hydrogel exhibited a compressive Young’s modulus of approximately 830 kPa and promoted osteogenic differentiation of bone marrow mesenchymal stem cells (BMSCs). In a rat calvarial defect model, near-complete bone repair was achieved after 12 weeks, with significantly higher BV/TV and BMD than control groups [28]. Jiang et al. loaded oncostatin M (OSM) into temperature/pH dual-responsive hydrogels [120]. Under an acidic microenvironment (pH = 6.5), the released OSM promoted angiogenesis and inhibited myocardial fibrosis, thereby improving cardiac function (Figure 5E). Al Enezy-Ulbrich et al. developed a microgel-reinforced fibrin composite hydrogel that exhibited excellent biocompatibility and superior mechanical properties [139]. This composite offers a novel concept for the functional customization of tissue engineering scaffolds. Beyond these examples, the integration of temperature/pH dual-responsive hydrogels with advanced fabrication techniques has enabled more sophisticated tissue engineering platforms. For cartilage repair, double-network microcrystalline cellulose hydrogels with high mechanical strength (compressive modulus ~1.2 MPa) and good biocompatibility have been developed to support chondrocyte proliferation and extracellular matrix deposition [5]. In the context of bone regeneration, injectable chitosan-based hydrogels with temperature/pH dual-responsiveness allow the minimally invasive delivery of osteogenic factors and stem cells, with the gelation kinetics tuned to match the inflammatory and remodeling phases of bone healing [25]. In summary, temperature/pH dual-responsive hydrogels serve not only as scaffolds for cell delivery but also as platforms for the controlled release of growth factors, thereby providing intelligent material support for tissue regeneration and repair.

### 4.4. Wound Healing

Wound healing is a complex process governed by the dynamic local microenvironment. However, conventional wound healing methods frequently prove inadequate as they lack the capacity to adapt to the dynamic wound conditions, such as temperature fluctuations, pH variations, and changes in exudate. To address this limitation, hydrogels have emerged as promising carriers that enable the spatiotemporally controlled release of therapeutic agents directly into the wound microenvironment. For instance, He et al. developed an in situ-forming smart wound dressing based on biomimetic cellulose nanocrystals, conferring responsiveness to temperature, pH, and NIR light [140]. This platform facilitates the co-delivery of doxorubicin and indocyanine green, conforms to irregular wound contours, and offers an optimal synergistic therapeutic strategy for healing wounds with postoperative drug-resistant bacterial infections. Similarly, Liu et al. developed a curcumin-loaded hydrogel that demonstrated significantly enhanced antibacterial efficacy against Escherichia coli and *Staphylococcus aureus* under simulated wound conditions [121], showcasing its potential as a platform for precise drug delivery and antibacterial therapy in wound healing. Haidari et al. developed a PNIPAM-co-PAAc-co-AgNPs hydrogel that responds to the alkaline pH (≥7.4) and elevated temperature of infected wounds. The hydrogel released 88% of silver ions within 24 h at pH 7.4, achieving over 95% bacterial killing in vitro. In a murine scald burn model, it reduced *Staphylococcus aureus* load by 1.58 log and achieved 75% re-epithelialization after 10 d [37]. This hydrogel responds to the elevated temperature and alkaline pH at the infection site, enabling on-demand AgNP release, effective eradication of infection, and accelerated wound healing (Figure 5F). Advancing the concept further, Hou et al. engineered a multifunctional microneedle patch for the treatment of chronic, infected diabetic wounds [141]. Xue et al. developed a temperature/pH dual-responsive hydrogel by integrating an antimicrobial nanoplatform into carboxymethyl chitosan and Pluronic F127 gels [142]. Animal experiments demonstrated excellent therapeutic efficacy in promoting the healing of *S. aureus*-infected wounds. The patch synergistically integrates antibacterial, anti-inflammatory, pro-angiogenic, and antioxidant functionalities. The temperature-responsive drug release facilitates biofilm penetration, while the underlying hydrogel maintains wound microenvironment homeostasis to markedly accelerate healing. Additionally, anthocyanins within the patch provide a pH-dependent colorimetric response, offering a visual indication of wound status. Consequently, temperature/pH dual-responsive hydrogels represent a widely explored category of intelligent dressings for advanced wound healing.

## 5. Conclusions and Outlook

Temperature/pH dual-responsive hydrogels, a prominent class of intelligent soft materials, exhibit synergistic responses to physiological stimuli, manifesting as reversible volumetric transitions between swollen and collapsed states. This synergistic responsiveness serves to optimize the performance of these materials, enabling sophisticated control over their behavior in complex biological environments. This review systematically outlines the primary synthesis strategies for these hydrogels, encompassing physical, chemical, and hybrid crosslinking approaches. The advantages and limitations of each method are compared and analyzed with respect to preparation efficiency, structural integrity, and biocompatibility. Furthermore, prevalent hydrogel structures are categorized, and the interrelationship between their structural design strategies, fabrication methods, and functional performance is thoroughly examined. Finally, a comprehensive overview of their principal biomedical applications is provided, spanning wound healing, tissue engineering and regenerative medicine, drug delivery, cancer therapy, as well as biosensing and diagnosis. Notably, these materials provide powerful strategies to enhance therapeutic precision while mitigating off-target and adverse effects, holding immense potential particularly in controlled drug delivery and targeted cancer therapy.

Despite the significant potential of temperature/pH dual-responsive hydrogels in biomedical applications, their widespread clinical translation is hindered by several challenges. Regarding scalability, many sophisticated structural design strategies—such as core–shell microspheres, bilayer actuators with graded crosslinking, and microfluidically generated particles—rely on low-throughput precision equipment, which presents a significant barrier to industrial-scale production. Regarding reproducibility, hybrid crosslinking strategies often suffer from poor batch-to-batch consistency due to variations in monomer purity, crosslinker distribution, and polymerization conditions, while the complexity of fabrication procedures further limits reproducible synthesis. Regarding biomedical applicability, chemically crosslinked hydrogels may introduce toxicity arising from residual crosslinking agents or degradation products, while their long-term biodistribution, chronic toxicity, and immunogenicity remain inadequately characterized. Moreover, appropriate sterilization methods for temperature-responsive hydrogels are underdeveloped, as conventional techniques (e.g., autoclaving, gamma irradiation, and ethylene oxide treatment) often compromise network integrity or leave toxic residues. Additionally, the insufficient sensitivity of the hydrogels may result in premature or incomplete drug release, and poor penetration into deep tumor tissue further limits the efficacy of cancer therapy. Collectively, these issues currently impede the widespread clinical adoption and translation of these promising smart materials.

To address the aforementioned limitations and advance the clinical translation of temperature/pH dual-responsive hydrogels, a multi-faceted optimization strategy is imperative. In terms of preparation methodologies, the development of biocompatible and green crosslinking technologies should be prioritized to minimize potential cytotoxicity risks. Furthermore, hybrid crosslinking processes must be simplified and standardized through systematic parameter optimization to ensure batch-to-batch reproducibility and scalability. At the structural design level, the response thresholds must be precisely tailored to match the unique demands of diverse physiological microenvironments. Modular biomimetic assembly offers a promising approach for constructing sophisticated architectures while improving compatibility with scalable manufacturing. For functional enhancement, strategies such as targeted peptide modification and nanocarrier integration can be employed to significantly improve tissue penetration depth, a critical factor for treating deep-seated tumors. Multi-level stimulus-response-release control systems should be implemented to prevent premature and incomplete drug release. Concurrently, integrated systems that embody reaction regulation, degradation matching, and functional synergy are necessary to ensure dynamic adaptation to the timelines of tissue regeneration and wound healing. Looking forward, interdisciplinary convergence is imperative. The integration of biosensing with image-guided technologies will facilitate real-time monitoring of in vivo hydrogel behavior. Simultaneously, establishing standardized performance evaluation systems and clear clinical translation pathways will lay the foundation for the widespread adoption of these smart materials in precision diagnosis and personalized medicine.

## Figures and Tables

**Table 1:** Fabrication strategies for temperature/pH dual-responsive hydrogels.

Crosslinking Type	Core Bonding Type	Advantages	Disadvantages	References
Chemical crosslinking	Static covalent bonds: C-C, amide, ester bonds Dynamic covalent bonds: hydrazone, imine (Schiff base), boronic ester, disulfide bonds	(1) Higher mechanical strength long-term stability (2) Tunable degradation	(1) Potential toxicity due to the crosslinking agent or degradation products (2) Static networks lack injectability and self-healing (3) Slow to moderate response	[15,30,53,54,55,56,58,59,60,61,62,63,68,69,70]
Physical crosslinking	Reversible non-covalent interactions: hydrophobic association, hydrogen bonding, electrostatic interactions, ionic coordination, chain entanglement	(1) Best injectability and biocompatibility (2) Fast response and self-healing ability	(1) Low mechanical strength (2) Poor long-term stability	[36,73,74,75,76,77,79,80]
Hybrid crosslinking	Covalent and non-covalent bonds: dual networks, nanoparticle-reinforced hybrid phases	(1) Balance of strength and injectability (2) Spatiotemporal control for complex microenvironments	(1) Most complex fabrication (2) Poor batch-to-batch reproducibility (3) Difficult to scale up	[52,82,83,84,85,87,88,90,91,92]

**Table 2:** Comparing the characteristics of the primary macroscopic structures of temperature/pH dual-responsive hydrogels.

Structure	Design Core and Principles	Key Functional Advantages	Major Limitations	References
Microsphere structure	A distinct functional carrier with a large specific surface area is provided by the microsphere unit containing the response function.	(1) Extremely quick speed of reaction (2) High load and quick material transfer	(1) The process of preparation is complicated. (2) Scaling production is challenging.	[31,94,95,96,97,98,99]
Core–shell structure	Shell gating, cascade response, and core protection are achieved through spatial partition.	(1) Excellent medication protection and capacity for tailored release (2) Stimulus sequential response cascade release	(1) The stages involved in preparation are numerous and laborious. (2) It is challenging to precisely manage its thickness and homogeneity.	[32,100,101,102,103]
Layered structure	The internal tension caused by the heterogeneous layers swelling differential is used to create the bending deformation.	(1) Excellent abilities for driving (2) High levels of functional integration	(1) The stability of the interlayer interface is poor. (2) High requirements for the preparation process.	[104,105,106,107,108,109,110,111]

**Table 3:** Comparison of key performance metrics of some temperature/pH dual-responsive hydrogels in drug delivery.

Material System	Drug Loaded	Key Performance Metrics	Stimulus Conditions	References
mPEG-PA-PLL hydrogel	Calcitonin	Encapsulation > 96%; release ~100% in 24 h	pH 6.8, 37 °C	[129]
Dual-responsive polymersomes	DOX + PTX	DOX release 60–70%, PTX 70–80% in 4 d; CI (MCF-7) = 0.72	pH 6.0, 45 °C	[118]
P(VCL-co-AA) nanogel	DOX	Loading efficiency 83%; IC50 lower than free DOX	pH 5.4, 40 °C	[136]
Gelatin-OSM hydrogel	PTX	63% release in 9 d; U87MG viability ~35%	pH 6.5, 37 °C	[42]
Magnetic nanocomposite particles (MNCPs)	MTX	87% release in 42 h; MCF-7 viability 28% (with hyperthermia)	pH 5.5, 42 °C	[103]

## Data Availability

No new data were created or analyzed in this study. Data sharing is not applicable to this article.
